# Improvements to Unsteady Pressure-Sensitive Paint Formulations

**DOI:** 10.3390/s25185892

**Published:** 2025-09-20

**Authors:** Sarah M. Peak, Daniel T. Reese, Kyle Z. Goodman, A. Neal Watkins

**Affiliations:** 1 NASA Langley Research Center, Hampton, VA 23666, USAanthony.n.watkins@nasa.gov (A.N.W.); 2 Analytical Mechanics Associates, Hampton, VA 23666, USA

**Keywords:** pressure sensitive paint, frequency response, titanium dioxide, luminescence, fluorescence, dispersant

## Abstract

**Highlights:**

**What are the main findings?**

- Hydrophobic TiO_2_ particles generate smoother and thinner uPSP coatings.
- Choice of dispersant can greatly affect the linearity of the pressure sensitivity.

**What is the implication of the main finding?**

- Even after degradation, one of two dispersants tested showed minimal loss of pressure sensitivity.
- Use of this dispersant showed pressure sensitivity was less dependent on temperature.
- New PSP formulations showed minimal change in frequency response.

**Abstract:**

Improvements to unsteady pressure-sensitive paint (uPSP) formulations have been realized by judicious selection of titanium dioxide (TiO_2_) particles and dispersant. Traditionally, uPSP formulations based on polymer/ceramic coating have been used in many wind tunnel test campaigns but suffer from photodegradation and changes in pressure sensitivity during the testing window. As such, this paper details the investigation of employing different grades of TiO_2_ particles and dispersants to achieve desirable characteristics such as coating properties, pressure sensitivity, frequency response and overall degradation. Employing hydrophobic TiO_2_ particles along with a high-molecular-weight acrylic co-polymer generated uPSP coatings with many desirable features, including smoothness, thickness, and pressure sensitivity. In addition, the pressure sensitivity of the coatings exhibited linear behavior, having very little dependence on temperature. Finally, the frequency response was characterized qualitatively, and all uPSP formulations tested exhibited response to pressure fluctuations up to 12 kHz.

## 1. Introduction

In the field of aerodynamics, the accurate determination of spatially continuous pressure distributions on surfaces is critical for understanding complex flow mechanisms and for comparison with computational fluid dynamics (CFD) predictions. This is especially true when these surfaces experience unsteady flow, such as turbulence, flutter, and buffeting. In most ground testing facilities, these measurements are currently made using conventional unsteady pressure transducers. While these instruments provide accurate pressure information, they only provide measurements at discrete points on the surface and integration of a sufficient number of these transducers on larger-scale models can be time- and labor-intensive, as well as costly. A potential solution to these issues can be found using the Pressure-Sensitive Paint (PSP) technique [1,2,3,4,5].

PSP [6,7,8,9,10] is an optical technique that is based on the oxygen sensitivity of luminescent probe molecules suspended in gas-permeable binder materials. With proper illumination, the probe molecules absorb a photon resulting in an excited energy state which relaxes to the ground state either by fluorescence/phosphorescence (radiative process) or non-radiative processes, such as conversion of the energy to heat or collisional quenching with another molecule. For PSP applications, oxygen acts as a collisional quencher, and this interaction with oxygen can be used to calculate pressure. The rate at which these two processes (radiative vs. non-radiative) compete is dependent on the concentration of oxygen present and can be described by the Stern–Volmer relationship [11]

(1) $$I_{0}/I=1+K_{\mathrm{SV}}\left(T\right)P_{O_{2}}$$

where I_0_ is the luminescence intensity in the absence of O_2_ (i.e., vacuum), I is the luminescence intensity at some partial pressure of oxygen (P_O2_), and *K_SV_* is the Stern–Volmer constant, which is dependent on temperature (T).

There are several issues with this relationship, especially in regard to wind-tunnel applications; first, it is a practical impossibility to measure *I*_0_ in a wind tunnel application. Second, the luminescent signal from the paint is not only a function of pressure; it also varies with factors such as illumination intensity, probe molecule concentration, and paint layer thickness. These spatial variations typically result in a non-uniform luminescence signal from the painted surface. The spatial variations are usually eliminated by taking a ratio of the luminescent intensity of the paint at the test condition with the luminescent intensity of the paint at a known reference condition– usually at wind-off. Thus, Equation (1) can be cast into a more suitable form

(2) $$I_{\mathrm{REF}}/I=A(T)+B\left(T\right){*P}_{O_{2}}$$

where *I_REF_* is the intensity at the reference conditions. The coefficients *A*(*T*) and *B*(*T*) are for a given PSP formulation and are usually determined beforehand using laboratory calibration procedures.

**Figure 1:**
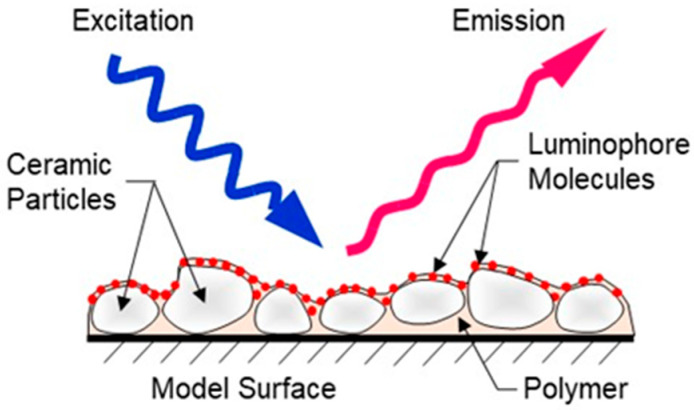
PSP formulation schematic.

Current PSP formulations typically have a slow response time (on the order of milliseconds to seconds) and are mainly used to measure static flows. With the improvement and development of camera and lighting technologies, the demand to measure unsteady flow phenomena has increased. Recently, unsteady PSP (uPSP) formulations applied using conventional spraying techniques have been developed that have response times up to 20 kHz [12]. These response times are possible due to the use of binders that have a higher oxygen diffusion rate than conventional PSP formulations. The uPSP technique has been used on a variety of models, including flexible rotor blades [13,14,15,16], rotating turbomachinery [17,18,19,20,21], and launch vehicles [22,23,24]. uPSP formulations are usually based on a polymer/ceramic binder system which consists of white pigment particles suspended in a polymer. This tends to result in a coating that is much rougher than more traditional PSP coatings but also provides a faster responding paint due to the increase pore size imparted by the ceramic pigment particle. When applying the luminescent dye onto a surface with large pore size, most of the dye molecules end up being on the surface making them easily accessible to oxygen. A graphical representation of these types of uPSP binders is shown in Figure 1 [21,22].

Although current uPSP formulations show great promise, there are some significant drawbacks that need to be addressed. One main drawback is the fast degradation of the uPSP paint. This is thought to be due to the presence of titanium dioxide (TiO_2_) which can act as a photocatalyst causing the photobleaching of the luminescent dye [25,26]. Several studies have investigated surface modifications to TiO_2_ particles that can aid in photocatalysis [27,28] or reduce this effect [29].

Previous studies have investigated different particles (mesoporous silicon dioxide [m-SiO_2_] [30,31], titanium silicon oxide [32], boron nitride [33], etc.); however, many exhibit drawbacks such as lower pressure sensitivity, less luminescence output, and cost factors (especially in the case of m-SiO_2_). Even using a “typical” TiO_2_ pigment particle can significantly affect the performance of a uPSP formulation. Manufacturers of TiO_2_ particles often produce several different grades that can have very different properties depending on surface treatment. The most common treatment of TiO_2_ is the addition of silica and/or alumina to the surface and some grades have a final organic coating applied at the end [34]. While the specific treatment of the TiO_2_ particles depends on the application, in general silica is added to form a shell around the TiO_2_ for UV resistance, alumina is added to reduce particle attraction (i.e., enhance dispersibility) and the organic treatment determines whether the particle is hydrophobic or hydrophilic. Recently, Kasai et al. [35] showed that uPSP formulations had the best adhesion when the polymer and TiO_2_ pigment had the same wettability (hydrophobic or hydrophilic). They used a hydrophobic polymer combined with hydrophobic TiO_2_ and the resulting uPSP formulation maintained similar response times.

This work investigates uPSP formulations employing a commonly used hydrophilic polymer. It also demonstrates that uPSP formulations with very different performance properties can be realized by judicious selection of the dispersant and the grade of TiO_2_ employed. The performance properties investigated here include brightness, film thickness and roughness, pressure sensitivity, time response, and degradation with time.

## 2. Materials and Methods

### 2.1. Materials

All reactions and solutions requiring water were performed using Milli-Q-grade deionized water (resistivity of 18.2 MΩ). Triflurotoluene was purchased from Aldrich. Different TiO_2_ grades were generously provided by Chemorous. 5,10,15,20-tetrakis(2,3,4,5,6-pentafluorophenyl)porphyrin-22,24-diide platinum(II) (PtTfPP) was purchased from Frontier Scientific. Rhoplex HA-8 was purchased from DOW. All chemicals were used as received.

### 2.2. Pressure-Sensitive Paint Formulations

All PSP experiments were performed on round aluminum plates (5 cm diameter, ~2 mm thick) on which the PSP was applied using an airbrush. Briefly, the coupons were first painted with a self-etching primer to aid in the paint adherence, followed by an epoxy base coat, which acts as an inert layer to screen the PSP from the self-etching primer and aluminum surface. On the epoxy layer, a white acrylic polymer/ceramic coating is applied. Binder solutions were made by mixing a slurry solution which consisted of 50 g TiO_2_, 30 mL of water, and dispersant (0.2 mL dispersant A or 0.5 mL dispersant B) and were mixed for 4 h using a roller mill. Dispersant A is a polyacid dispersant supplied in the ammonia form at 35% solids and recommended for latex formulations. This dispersant was used for the hydrophilic grades of TiO_2_ only since it was ineffective with the hydrophobic grades. Dispersant B is a high-molecular-weight acrylic co-polymer specifically designed to disperse TiO_2,_ Fe_2_O_3_, and other inorganic pigments. After mixing, the polymer (4 wt% based on the slurry, 3.2 g) was added. For these experiments, the polymer is an acrylic polymer that is self-crosslinking (Rhoplex HA-8 from Dow Chemicals). This polymer/ceramic coating is the basis for the PSP binder and has been used previously in wind tunnel tests [13,15,16,22,23,24]. The white binder serves as a matrix for the PtTfPP luminophore as well as an excellent scattering surface to direct more of the emission intensity to the camera. Finally, an overspray solution containing PtTfPP in triflurotoluene was applied to achieve a pale pink color. The same amount of PtTfPP was applied to each sample for a good comparison between samples. All applications were done using a siphon feed spray gun and allowed to dry in air for 1 h before testing.

### 2.3. Measurements

Thickness measurements were tested using an electronic thickness gauge (ETG2, Pro Motor Car, Inc., Costa Mesa, CA, USA) that has a stated accuracy of +/− 1%. Roughness measurements were acquired using a portable Mitutoyo Surftest SJ-210. All measurements were averaged using at least 10 different samples and reported as R_a_.

Static PSP performance was tested using a commercially available calibration system (Innovative Scientific Solutions, Inc. Dayton, OH, USA) that simulates pressure by controlling the oxygen and nitrogen levels in a small calibration chamber. Furthermore, the calibration chamber is equipped with a thermally controlled sample stage to control temperature. The calibration chamber can simulate pressures from 0 to 400 kPa and control temperature from 0 to 70 °C with an accuracy of 0.1 °C. Illumination of the PSP was accomplished using an LED lamp emitting at 400 nm (full-width at half-maximum of 20 nm) and emission was collected using a CCD camera (1600 × 1200 pixels, 12-bit digital resolution). An optical filter (650 nm with a bandwidth of 40 nm) was mounted in front of the CCD camera lens to discriminate between the emission light and the excitation light.

**Figure 2:**
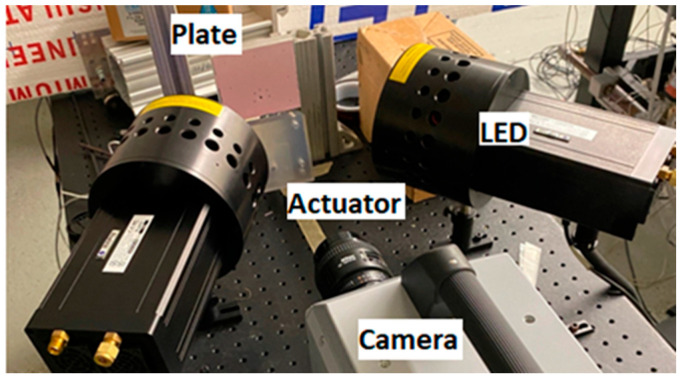
Setup for frequency response testing using fluidic actuator.

The frequency response of the PSP formulations was evaluated using specially designed sweeping jet actuators employing nitrogen gas. Nitrogen was employed to aid in visualization of the PSP response. The design and performance characteristics of the actuators are described in Melton et al. [36]. For these experiments, aluminum plates (80.3 mm × 100 mm × 20 mm thick) equipped with a dynamic pressure transducer were employed, and the PSP formulations were applied using the techniques described above. LED illumination was again used to provide 400 nm light and images were acquired using a high-speed camera (Photron SA-Z) collecting images at a frame rate of 25,000 fps. Imaging was performed on an area approximately 38 mm × 38 mm, resulting in a resolution of approximately 27 pixels/mm. The dynamic pressure transducer data acquisition rate was also set to 25,000 Hz. The experimental setup of the actuator and equipment is shown in Figure 2. For this work, two different actuator designs were employed. The primary difference between the two is the planform size of the actuator such that they exhibit different frequency behavior controlled by the pressure ratio across the actuator and the length of the feedback channel. Thus, one actuator (MK7) could provide frequency content with a main frequency of 1.47 kHz with several higher harmonics, and the other actuator (MK6) can provide frequency content at 3.9 kHz also with several harmonics. Both actuators could provide harmonic frequencies up to approximately 12 kHz.

## 3. Results

### 3.1. General Coating Properties

For the optimization of the uPSP, five different TiO_2_ grades and two dispersants were investigated. The nature of the TiO_2_ (hydrophobic vs. hydrophilic) also somewhat controlled the type of dispersant that is used. Dispersant A is a polyacid dispersant supplied in the ammonia form at 35% solids and recommended for latex formulations. This dispersant was used for the hydrophilic grades of TiO_2_ only since it was ineffective with the hydrophobic grades. Dispersant B is a high-molecular-weight acrylic co-polymer specifically designed to disperse TiO_2_, Fe_2_O_3_, and other inorganic pigments. All grades of TiO_2_ were capable of being dispersed with this material. The overall general properties of the coatings were affected by both the type of dispersant used and the nature of the TiO_2_. The specific formulations tested as well as the measured thickness and roughness of the coatings are shown in Table 1. All formulations detailed here showed excellent adhesion regardless of which TiO_2_ grade was used.

Table 1 summarizes that the physical properties of the uPSP formulations (defined by the thickness and roughness of the films made from the different formulations) do not have a significant dependence on the additives to the TiO_2_ pigment (silica and alumina). In general, the water solubility of the TiO_2_ drives this, with hydrophobic TiO_2_ generally producing thinner and smoother coatings. However, for the hydrophilic TiO_2_ grades, smoother and slightly thinner films can be realized by using dispersant B. This is evidenced as TiO_2_ formulations 4 and 5 use the same type of TiO_2_ as formulations 2 and 3, respectively, with the only difference being the type of dispersant employed. From these data, it is shown that dispersant B along with the hydrophobic grade of TiO_2_ generally yields a smoother, thinner coating.

### 3.2. uPSP Calibration Performance

**Figure 3:**
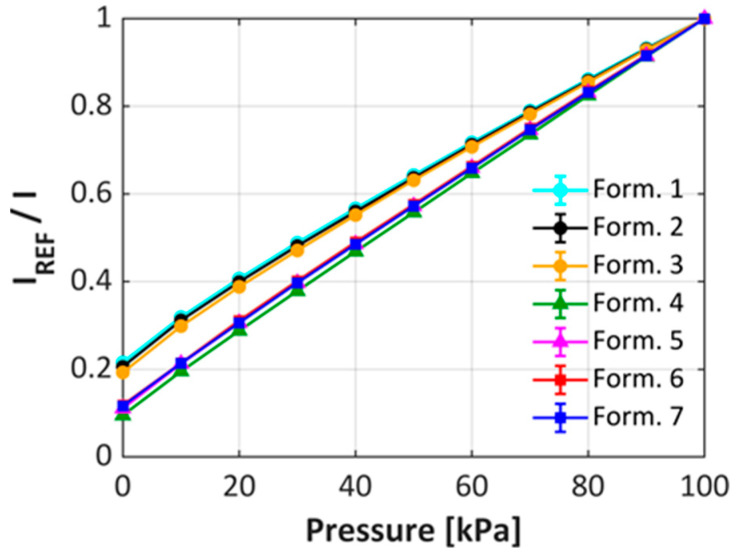
Pressure sensitivity of uPSP formulations 1–7. Error bars are present but contained inside the points.

As described in Section 2.3 above, the pressure sensitivity of the uPSP formulations were evaluated in a calibration chamber employing mixtures of nitrogen and oxygen. For each of the formulations listed in Table 1 above, the pressure sensitivity was obtained as the simulated pressure was increased from vacuum (no oxygen) to 100 kPa (20.9% oxygen) and the results are shown in Figure 3. It is readily apparent that there are two distinct shapes to the curves that are dependent only on the type of dispersant used. For those formulations employing dispersant B (4–7), both the hydrophilic and hydrophobic TiO_2_ displayed very linear pressure sensitivity, while those using dispersant A (1–3) resulted in a non-linear curve and shows a lower overall sensitivity. Previous studies have suggested that the non-linearity of the pressure sensitivity is due to the PtTfPP molecules residing in different environments in the coating [37,38]. While it can be inferred that this behavior may be only due to the use of dispersant B, it should be emphasized that the films from hydrophobic TiO_2_ particles cannot be constructed using dispersant A. However, for hydrophilic TiO_2_ formulations, the choice of the correct dispersant has significant effect on its pressure sensitivity.

**Figure 4:**
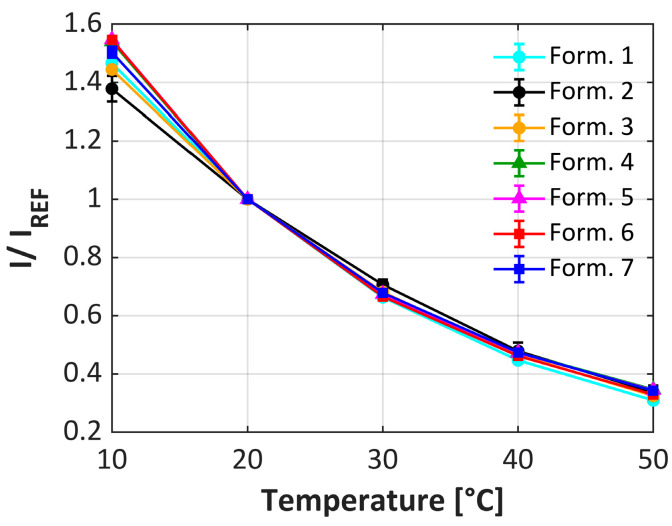
Temperature sensitivity of uPSP formulations 1–7. Error bars are present but contained inside the points.

**Figure 5:**
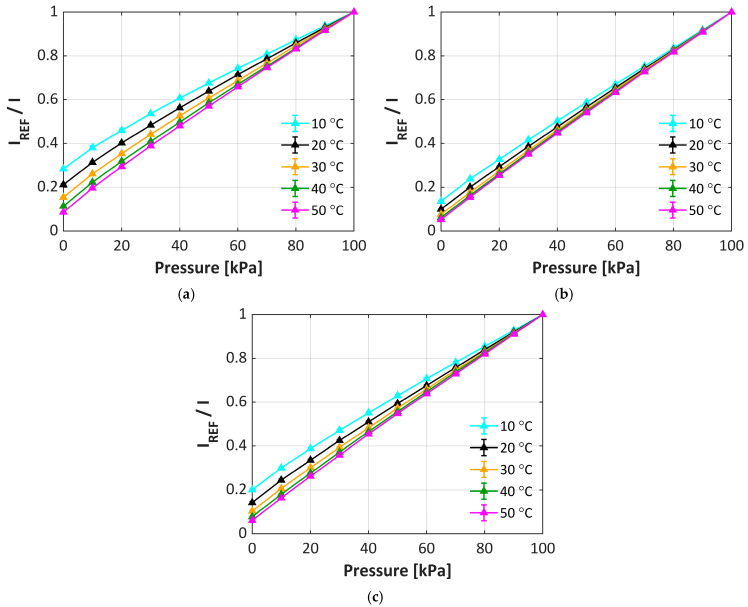
Pressure sensitivity at different temperatures for formulation 2 (**a**), 4 (**b**), and 7 (**c**).

When investigating the effect of temperature on the performance of the uPSP formulations, Figure 4 shows that the temperature sensitivity is relatively independent of both TiO_2_ grade and dispersant resulting in a temperature sensitivity of approximately 1.6%/°C, which is consistent with previously published results [12]. However, the dependence of the pressure sensitivity on temperature behaves very differently. For clarity, only a hydrophilic TiO_2_ formulation using both dispersant A (formulation 2) and dispersant B (formulation 4) and a hydrophobic TiO_2_ formulation (formulation 7) were compared, but similar trends were observed with all formulations. For formulation 2 (Figure 5a), there is a significant effect on the pressure sensitivity depending on the temperature. At lower temperatures, the pressure response is non-linear and as the temperature is increased, greater linearity is seen. In addition, the response to the pressure significantly increases from 0.7%/kPa to 0.9%/kPa (approximately 30% increase) as the temperature increases. With the hydrophilic TiO_2_ with dispersant B (formulation 4, Figure 5b), this behavior reverts to the linear behavior over all temperatures (similar to Figure 3) and the overall change in the pressure response as a function of temperature decreases (from 0.85%/kPa at 10 °C increasing to 0.94%/kPa at 50 °C, only approximately 10% increase). For the hydrophobic TiO_2_ formulation (formulation 7, Figure 5c), similar trends as formulation 4 are seen. Again, this is consistent with the choice of dispersant controlling much of the performance of the respective formulations.

### 3.3. Degradation

Because of the very high oxygen diffusion rate in most uPSP formulations as well as the presence of pigment materials such as TiO_2_, degradation of the uPSP formulation is generally expressed in terms of photodegradation, or loss of luminescence signal that occurs during illumination of the uPSP layer. In most cases where uPSP is employed, this is often mitigated by careful design of data acquisition, such as only illuminating the sample briefly for measurements. Furthermore, in the majority of wind tunnel facilities where this technique is employed, the oxygen sensitive dye is often reapplied by simply overspraying with the dye solution (as described in Section 2.2 above). For example, employing this type of careful design and allowing for overspraying, Sellers et al. were able to collect data on a large model over several days [22].

However, there are facilities and cases in which accessing the uPSP-coated surface for reapplication of the luminescent dye is problematic due to a variety of reasons (e.g., operating in a non-air-based fluid). There can also be long delays after uPSP application due to tunnel operating procedures. In these cases, degradation can also occur even in the absence of light, which was shown by Wu et al. [39]. Therefore, a study of the degradation of the formulations was carried out to determine if the overall life expectancy of the uPSP coatings could be extended by careful selection of the pigment grade and dispersants.

To determine the rate of degradation of the uPSP samples, R_d_ is defined as the rate of degradation of the luminescence intensity per day over the course of four days. Samples were tested in the calibration chamber and then stored in a dark box without any environmental controls. Each day, the coupon was tested for the luminescence intensity as well as a pressure response calibration. The degradation rate was determined using a similar method described by Egami et al. [40]:

(3) $$R_{d}\;=\;-\;(1\;–\;\frac{I_{t=4\;days}}{I_{inital}})\;\frac{1}{4}\;\times \;100\;[\%/day]$$

where *I_t=initial_* and *I_t=_*_4_* _days_* are the luminescence intensities at time 0 and after 4 days (96 h), respectively. Four samples of each formulation were tested to determine the average degradation rate, and the results are listed in Table 2. This shows that the uPSP formulations employing dispersant B (4–7) have significantly smaller degradation rates compared with uPSP formulations made using dispersant A (1–3). The grade of TiO_2_ and the particle size have little effect on the degradation rate, with little difference in the degradation rate between hydrophilic and hydrophobic particles in formulations using dispersant B.

**Figure 6:**
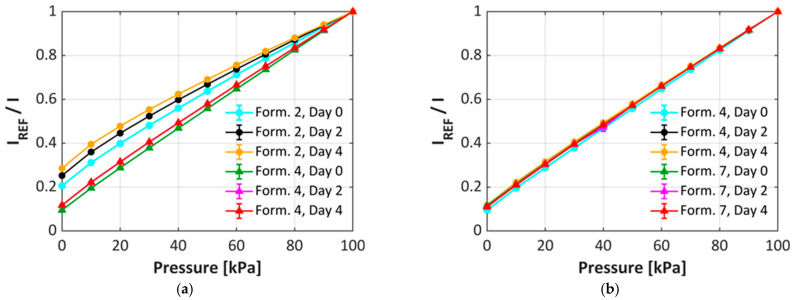
(**a**) uPSP pressure sensitivity at day 0, day 2, and day 4 for different dispersants A (Formulation 2) and B (Formulation 4); (**b**) uPSP pressure sensitivity at day 0, day 2, and day 4 for hy-drophilic (Formulation 4) and hydrophobic (Formulation 7) grades of TiO_2_.

In addition to the rate of degradation, the pressure sensitivity at 20 °C was investigated each day. For simplicity, only the initial, day 2, and day 4 pressure sensitivities are shown. Figure 6a is a comparison of formulations 2 and 4, which are formulations made from a hydrophilic TiO_2_ grade and using either dispersant A (formulation 2) or dispersant B (formulation 4). This data shows that the choice of dispersant has a great impact on the change in pressure sensitivity over time. Formulation 2 exhibits a significant decrease in both pressure sensitivity and linearity over time. In contrast, formulation 4 exhibits very little change in the linearity or sensitivity over the same time frame. This shows that by using dispersant B, the uPSP formulation can be stored up to four days with little change to the pressure sensitivity. A comparison of formulations made using the same dispersant (dispersant B) but using either hydrophilic (formulation 4) or hydrophobic (formulation 7) TiO_2_ particles are shown in Figure 6b. In this case, there is little change in the linearity of sensitivity of the formulations, thus indicating that the choice of dispersant can be critical for optimizing the overall performance of the uPSP formulation.

### 3.4. Frequency Response

The previous results have shown that several factors need to be considered when designing uPSP formulations and these greatly affect the morphology of the film, pressure sensitivity, and degradation rate of the coating. In general, the best films can be created using hydrophobic TiO_2_ grades with dispersant B. However, there is still one factor that must be considered and that is the frequency response of the uPSP. To qualitatively determine the frequency response of the uPSP formulations, an oscillating pressure signal was generated by a sweeping jet actuator positioned tangent to the PSP surface, as described in Section 2.3 above. After acquiring both image data and dynamic pressure transducer data at 25 kHz, raw video files from the high-speed camera were processed to provide a measure of how the uPSP formulation responded to the sweeping jets. This was done by first averaging the luminescence intensity in a 60-by-60 pixel region near the pressure transducer. The pressure–time history was then used to calculate the normalized power spectra followed by smoothing using a 20 Hz moving average filter.

**Figure 7:**
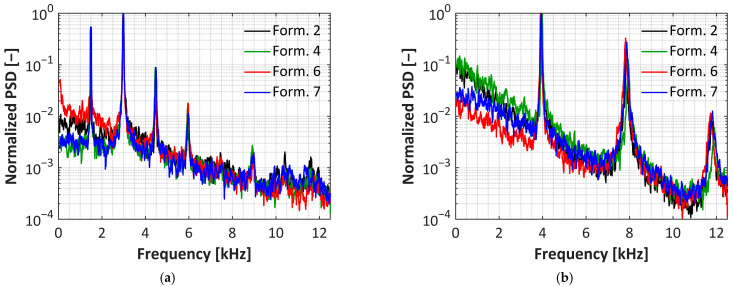
Frequency response of uPSP formulations using the MK 7 (**a**) or MK 6 (**b**) sweeping jet actuator.

The normalized power spectra for formulations 2, 4, 6, and 7 are shown in Figure 7. These show that all of the uPSP formulations generally show the same response to the jet actuators regardless of TiO_2_ grade or dispersant type. Oscillator MK 7 (Figure 7a) clearly shows a main frequency peak at 1.47 kHz followed by resonance signals at 2.9, 4.47, and 5.9 kHz. Although response is seen above 6 kHz, it is difficult to distinguish the details. To observe frequencies over 6 kHz, oscillator MK 6 (Figure 7b) was used and clearly demonstrates frequencies at 12 kHz. This oscillator had a main frequency peak at 3.9 kHz followed by resonances at 7.8 and 11.8 kHz. Using these two oscillators demonstrated that all uPSP formulations tested can respond to at least 12 kHz fluctuations. However, this is more of a qualitative technique and additional dynamic measurements to determine roll-off frequencies will need to be performed for a full characterization of the formulations.

## 4. Conclusions

Numerous studies have investigated ways to improve uPSP coating that can be used in a variety of wind tunnels. Currently, there are a few major drawbacks to uPSP such as non-ideal physical properties (coatings are inherently rough), a high rate of photodegradation, a non-linear pressure response, and a change in pressure response with respect to temperature. This work demonstrated that by using hydrophobic TiO_2_ particles, a smoother, thinner uPSP coating could be generated which can help minimize the intrusive effects of uPSP coating onto a wind tunnel model.

A high-molecular-weight acrylic copolymer dispersant (dispersant B) was used to generate uPSP formulations with desirable characteristics related to performance. First, the pressure response was higher as compared to formulations using dispersant A. In addition, the linearity of the pressure response was greatly improved resulting in easier calibration calculations. The overall temperature response for all formulations tested were similar to traditional uPSP samples at 1.6%/°C. However, major differences were seen with uPSP formulations using dispersant B which showed pressure response that was essentially independent of temperature. The formulations employing dispersant A displayed pressure responses that changed drastically with temperature.

All uPSP formulations tested underwent degradation but the formulations employing dispersant B displayed less degradation compared to those using dispersant A, 5%/day vs. 13.5%/day, respectively. Upon degradation, uPSP formulations employing dispersant A showed a major change in the shape and magnitude of the pressure response. However, by using dispersant B, the change in pressure response with degradation could be almost eliminated. This lower degradation and minimal pressure change upon degradation will be beneficial in wind tunnels that do not allow for tunnel access on a daily basis.

To be considered an unsteady paint, uPSP formulations must also have a fast response time. By using two different oscillators, it was shown that all uPSP formulations tested qualitatively showed the ability to respond at rates up to 12 kHz. Further investigations need to be performed to quantitatively determine the frequency response of new uPSP formulations.

## Figures and Tables

**Table 1:** uPSP formulations, physical properties of the TiO_2_ particles used, and physical properties of the uPSP coatings.

Formulations	1	2	3	4	5	6	7
TiO_2_ grade	I	II	III	II	III	IV	V
Hydrophilic (+) or Hydrophobic (−)	+	+	+	+	+	−	−
Silica wt%	0	3	6.5	3	6.5	0	3.5
Alumina wt%	1.7	2.5	4.0	2.5	4.0	1.7	3.3
Size of TiO_2_ particles (μm)	0.29	0.36	0.5	0.36	0.5	0.22	0.31
Dispersant	A	A	A	B	B	B	B
Roughness (R_a_, μm)	6.0 ± 1.3	6.5 ± 1.3	6.4 ± 2.4	3.3 ± 0.8	4.1 ± 0.1	1.0 ± 0.4	3.9 ± 0.5
Thickness (µm)	55.9 ± 17.8	40.6 ± 17.8	66.0 ± 27.9	50.8 ± 17.8	30.5 ± 10.2	27.9 ± 7.6	27.9 ± 17.8

**Table 2:** Degradation rates for uPSP formulations 1–7.

TiO_2_ Formulations	1	2	3	4	5	6	7
TiO_2_ grade	I	II	III	II	III	IV	V
Hydrophilic (+) or Hydrophobic (−)	+	+	+	+	+	−	−
Dispersant	A	A	A	B	B	B	B
Degradation rate (%/day)	−12.2 ± 6.2	−13 ± 3.9	−13.5 ± 4.6	−7.3 ± 3.5	−7.8 ± 3.1	−5 ± 1.2	−7.6 ± 2.9

## Data Availability

Selected data can be provided upon request.

## Abbreviations

The following abbreviations are used in this manuscript:

PSP	Pressure-Sensitive Paint
uPSP	unsteady Pressure-Sensitive Paint
PtTfPP	5,10,15,20-tetrakis(2,3,4,5,6-pentafluorophenyl)porphyrin-22,24-diide platinum(II)
m-SiO_2_	mesoporous silicon dioxide
TiO_2_	titanium dioxide
CCD	Charge-coupled device
mm	millimeter
nm	nanometer
Hz	Hertz
kHz	kilohertz
kPa	kilopascal
fps	frames per second
